# Supplementary material for: Structural basis for the unique molecular properties of broad-range phospholipase C from Listeria monocytogenes
Source: Nat Commun. 2023 Oct 14;14:6474. doi: 10.1038/s41467-023-42134-4 (PMC10576769; doi:10.1038/s41467-023-42134-4)
Supplement: Supplementary file 1 — Supplementary information [file 41467_2023_42134_MOESM1_ESM.pdf]

Structural basis for the unique molecular properties of broad-range phospholipase C from *Listeria monocytogenes*

Nejc Petrišič[1,2], Maksimiljan Adamek[1], Andreja Kežar[1], Samo B. Hočevar[3], Ema Žagar[4], Gregor Anderluh[1] and Marjetka Podobnik[1*]

[1]Department of Molecular Biology and Nanobiotechnology, National Institute of Chemistry Ljubljana, Slovenia

[2]PhD Program 'Biosciences', Biotechnical Faculty, University of Ljubljana, Ljubljana, Slovenia

[3]Department of Analytical Chemistry, National Institute of Chemistry Ljubljana, Slovenia

[4]Department of Polymer Chemistry and Technology, National Institute of Chemistry Ljubljana, Slovenia

*Corresponding author: marjetka.podobnik@ki.si

Supplementary information file contains:

1. Figures 1-20,
2. Tables 1-3,
3. Supplementary References

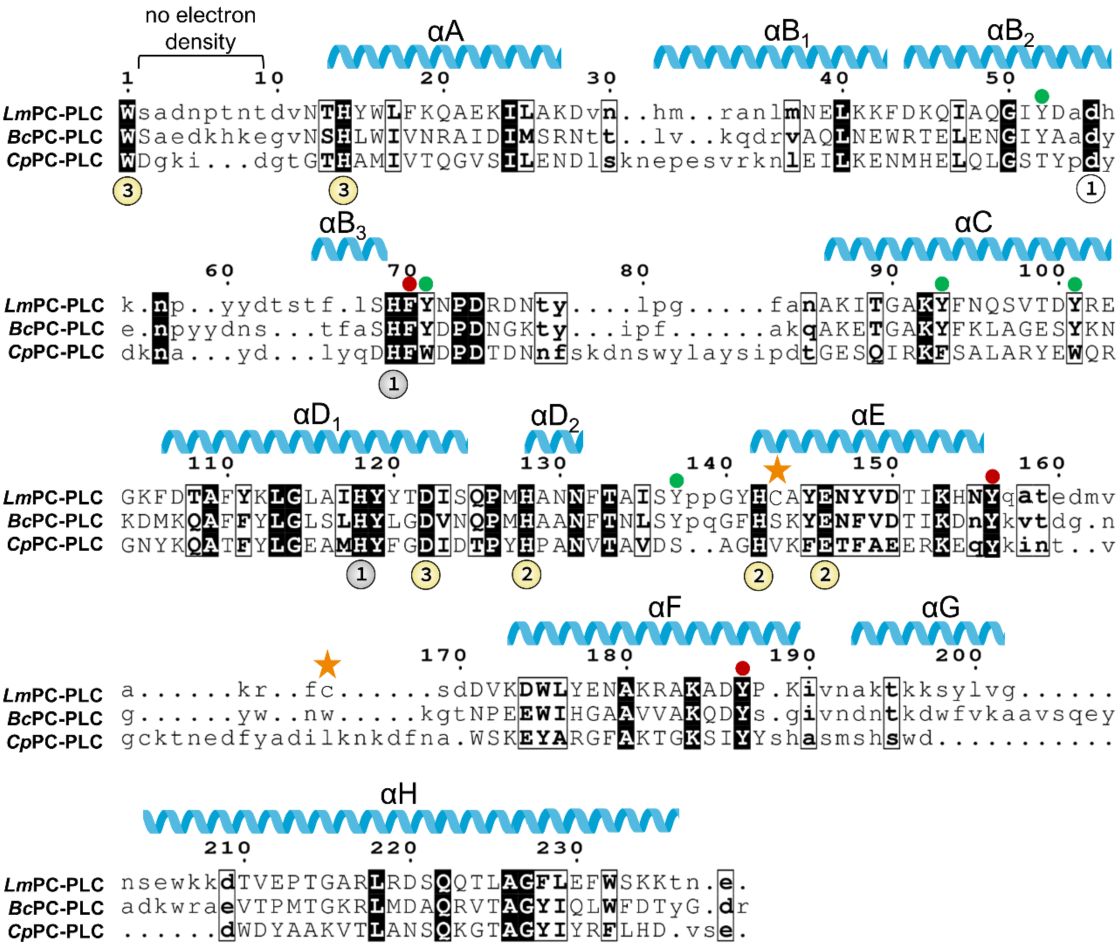

Supplementary Fig. 1: Structure based sequence alignment of the bacterial PC-PLC homologs. Structures of *Lm*PC-PLC (PDB-ID: 8QCM), *Bc*PC-PLC (PDB-ID: 1AH7) and the catalytic phospholipase domain of *Cp*PC-PLC (PDB-ID: 1CA1) were aligned using PDB eFOLD[1] with manual correction based on the alignment in Pymol2[2]. Sequentially conserved residues are in filled black frames and partially conserved with open white frames. Structurally aligned residues are shown in uppercase, residues with divergent positions in lowercase. The conserved residues, coordinating the active site metal ion(s), are marked by numbered circles: numbers (1, 2 or 3) indicate, which active site metal ion (Zn1, Fe2 or Fe3) is coordinated by these residues. Orange stars show the positions of the two unique cysteine residues in *Lm*PC-PLC (both replaced by Ser in *Lm*PC-PLC$^{C143S+C168S}$, of which crystal structure was solved). Red dots above the marked residues indicate structurally conserved surface exposed Tyr and Phe residues between all three homologues. Green circles show those conserved only among *Lm*- and *Bc*PC-PLC (see Supplementary Fig. 8c). Figure was created in Endscript 2[3], secondary structure representation is based on Pymol2 (v 2.5.2)[2].

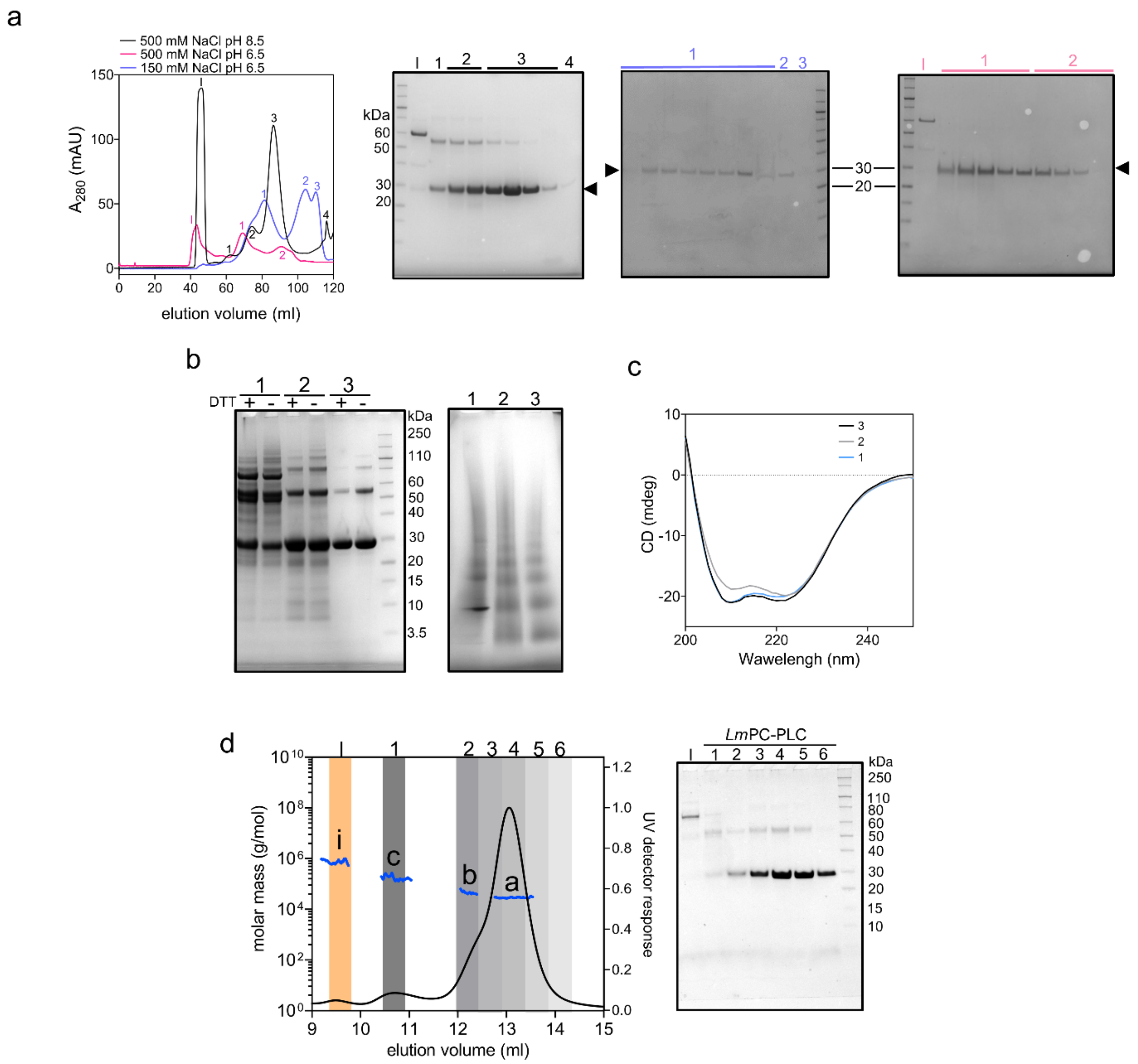

Supplementary figure 2: Purification and oligomerization of *Lm*PC-PLC a Left: SEC analysis of WT *Lm*PC-PLC at different salt concentrations and pH values (Tricorn 16/600 Superdex 75 pg), Right: SDS-PAGE of WT *Lm*PC-PLC after SEC in 500 mM NaCl, pH 8.5 (black lines); indigo: SEC using 150 mM NaCl, pH 6.5; pink: SEC using 500 mM NaCl, pH 6.5. *Lm*PC-PLC bands are marked with black triangles. **b** Left: SDS-PAGE of SEC peaks 1, 2, and 3 (from the panel (a), black curve) with or without DTT (20 mM). Right: Blue native-PAGE of SEC peaks 1, 2, and 3 (panel (a), black curve) without DTT. **c** CD spectra of WT *Lm*PC-PLC, SEC peaks 1, 2, and 3 (0.1 mg/ml) in 10 mM sodium phosphate (pH 7.4). **d** Left: SEC-UV chromatogram of *Lm*PC-PLC (black curve) obtained on a Tricorn 10/300 Superdex 75 GL column together with molar mass as a function of elution volume (blue lines). ‘a’ indicates monomers, ‘b’ dimers, ‘c’ higher order oligomers of *Lm*PC-PLC and ‘i’ impurity. Right: SDS-PAGE of the fractions marked on the chromatogram on the left. Buffer used was 20 mM Tris-HCl pH 8.5, 500 mM NaCl. Right: SDS-PAGE of *Lm*PC-PLC fractions from SEC-MALS run.

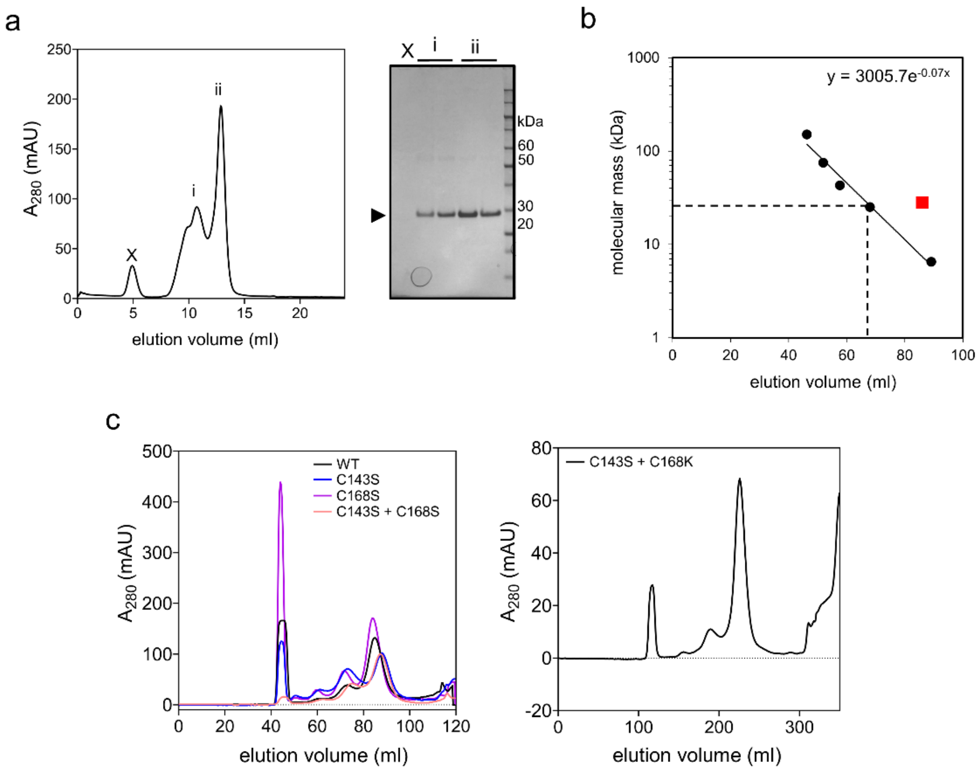

Supplementary figure 3: *Lm*PC-PLC oligomers and cysteine mutants a Left: SEC analysis of WT *Lm*PC-PLC monomer (peak 3 from the panel (a)) SEC re-run in 20 mM Tris-HCl pH 8.5, 500 mM NaCl, using Tricorn 10/300 Superdex 75 GL. Right: SDS-PAGE gel of *Lm*PC-PLC of the SEC re-run, bands representing *Lm*PC-PLC marked with a black triangle. **b** Calibration curve using Tricorn 16/600 Superdex 75 pg (20 mM Tris-HCl pH 8.5, 500 mM NaCl). Protein standards (black circles): 150 kDa: IgG, 75 kDa: transferrin, 43 kDa: ovalbumin, 25 kDa: scFvs, 6.5 kDa: aprotinin. Red square: the non-standard elution of 28 kDa *Lm*PC-PLC. Dashed line show expected elution volume of 28 kDa globular protein (see Fig 1a). **c** Left: SEC analysis of *Lm*PC-PLC cysteine mutants (Tricorn 16/600 Superdex 75 pg column, 20 mM Tris-HCl pH 8.5, 500 mM NaCl). Right: *Lm*PC-PLC$^{C143S+C168K}$ was analyzed on Tricorn 26/600 Superdex 75 pg column, therefore shown separately. Source data are provided as a Source Data file.

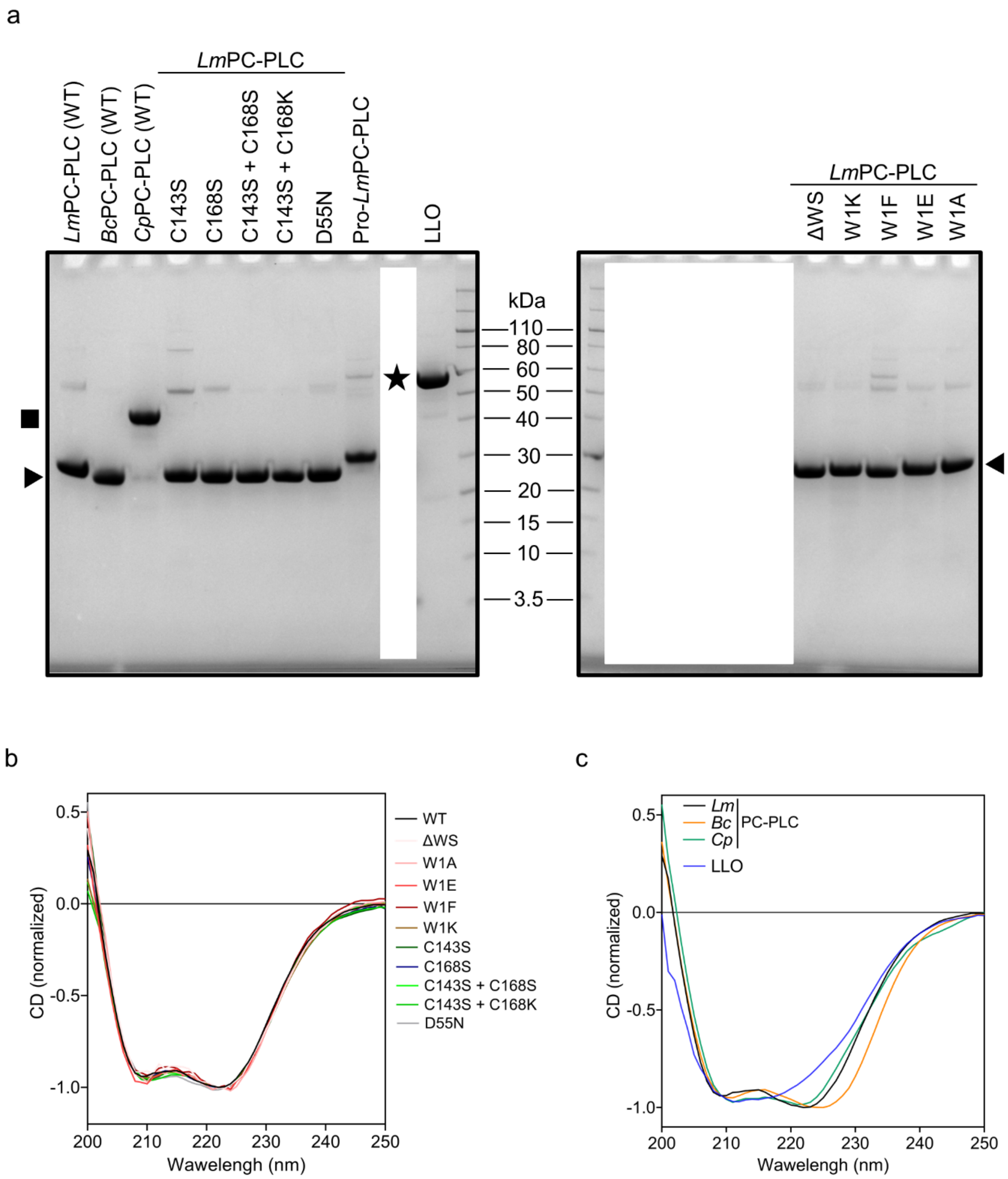

Supplementary figure 4: SDS-PAGE and circular dichroism (CD) spectra of proteins used in the study. a SDS-PAGE of purified proteins used in this study. The black triangle marks *Lm*PC-PLC and *Bc*PC-PLC, the black square marks *Cp*PC-PLC, and the black star marks LLO. Other samples in the gel were not relevant for this study and were omitted from the figure (white rectangle). **b** CD spectra (normalized to the wavelength with highest signal in each curve) of *Lm*PC-PLC WT and mutants (0.2 mg/ml) at pH 7.4. **c** CD spectra of the wild-type form of the three PC-PLC homologs and LLO (0.2 mg/ml) at pH 7.4. Source data are provided as a Source Data file.

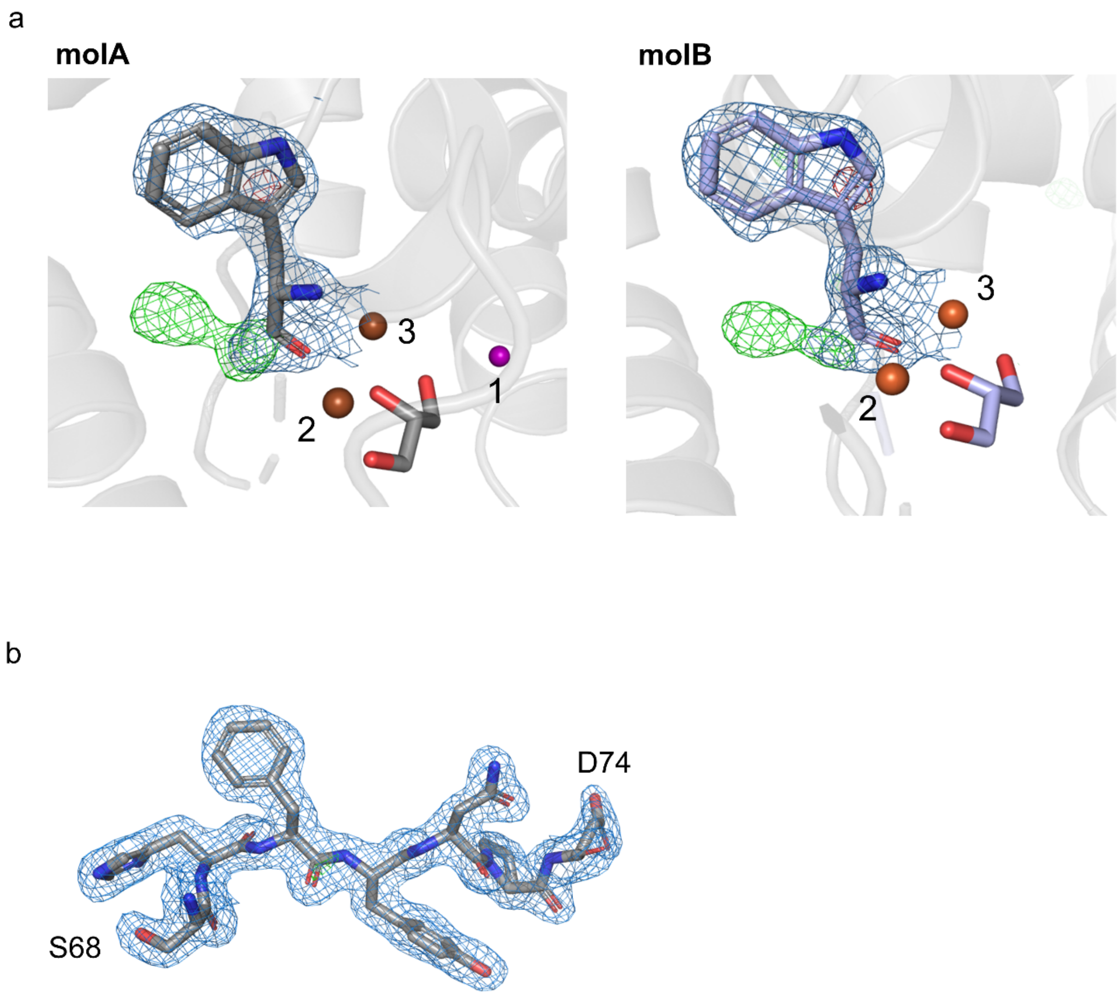

Supplementary figure 5: Representative electron density in the crystal structure of *Lm*PC-PLC. **a** Electron density of W1 (left: molA, right: molB). Zn ion (violet) and Fe ions (brown) are shown as spheres. W1 and glycerol are shown in sticks. **b** Structural detail (molB) of the region S68-D74. 2mFo-DFc electron density is contoured at 1 σ (blue) and mFo-DFc electron density at +3.0 σ (green) and at –3.0 σ (red). The green difference density in (a) indicates that the polypeptide chain continues after W1, however, residues S2-T9 were not defined by the electron density map.

a

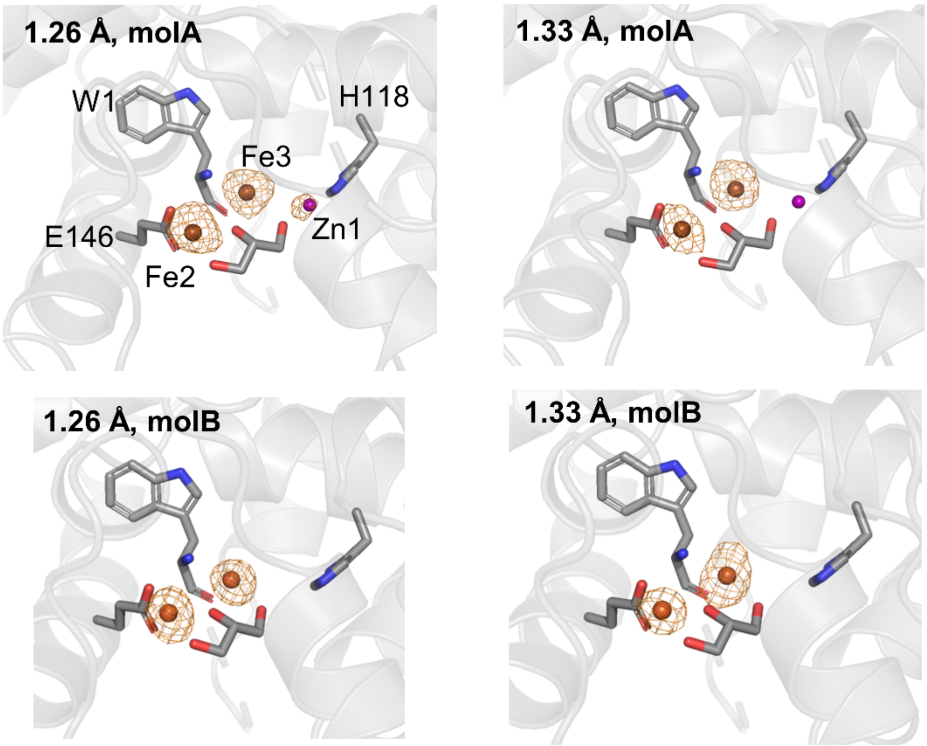

b

	1.26 Å	1.33 Å
Fe3 (molA)	10.5	10.5
Fe2 (molA)	13.5	5.5
Fe3 (molB)	12.7	12.8
Fe2 (molB)	16.5	9.8

	1.26 Å	1.33 Å
Zn1 (molA)	4.9	-
Zn1 (molB)	-	-

c

ICP-OES

	sample
protein (S)	35.70 ± 1.31 nmol

ICP-MS

	sample	ion to protein ratio
Fe	50.88 ± 0.87 nmol	1.43
Zn	0.36 ± 0.023 nmol	0.01

Supplementary figure 6: Identification of the active site metal ions in *Lm*PC-PLC using anomalous difference maps, ICP-OES and ICP-MS. a Anomalous difference map of the active sites (molA and molB) from data collections of a same crystal at two wavelengths (1.26 Å and 1.33 Å). Map contour level (σ) is 3.0. **b** Table of anomalous signal strength at active site metal positions 1, 2 and 3. The strength is described as the σ level of the anomalous difference map, where the density is not seen anymore. **c** Protein and metal content as determined by ICP-OES and ICP-MS analysis, respectively. No other metal ions were enriched in the *Lm*PC-PLC sample. Source data are provided as a Source Data file.

a b c

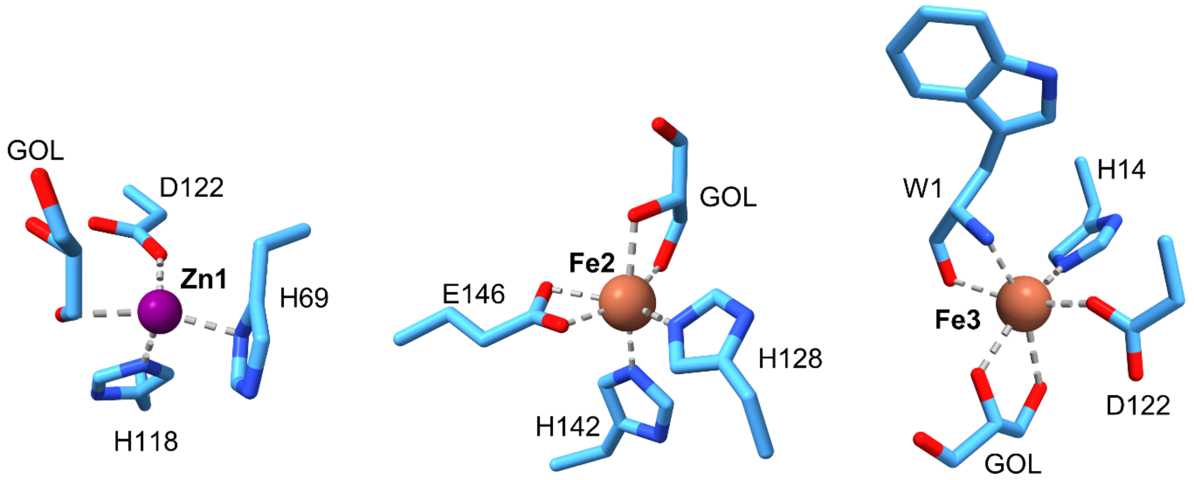

d

ion	ligand	d (Å)
ZN1	GOL (O1)	2.6
ZN1	D122 (OD2)	1.9
ZN1	H69 (ND1)	2.3
ZN1	H118 (NE2)	2.2

ion	ligand	d (Å)
FE2	E146 (OE2)	2.1
FE2	E146 (OE1)	2.5
FE2	H142 (NE2)	2.2
FE2	H128 (NE2)	2.1
FE2	GOL (O2)	2.4
FE2	GOL (O3)	2.0

ion	ligand	d (Å)
FE3	W1 (O)	2.5
FE3	W1 (N)	2.3
FE3	H14 (NE2)	2.2
FE3	D122 (OD1)	2.2
FE3	GOL (O1)	2.6
FE3	GOL (O2)	2.4

Supplementary figure 7: Coordination of the active site metal ions in *Lm*PC-PLC. a Zn1, **b** Fe2, **c** Fe3 coordination in the active site of molA of *Lm*PC-PLC. Coordination at position F2 and F3 in molB are the same as in molA. Glycerol (GOL) is shown in sticks. **d** Table of the distances between ions and their ligands.

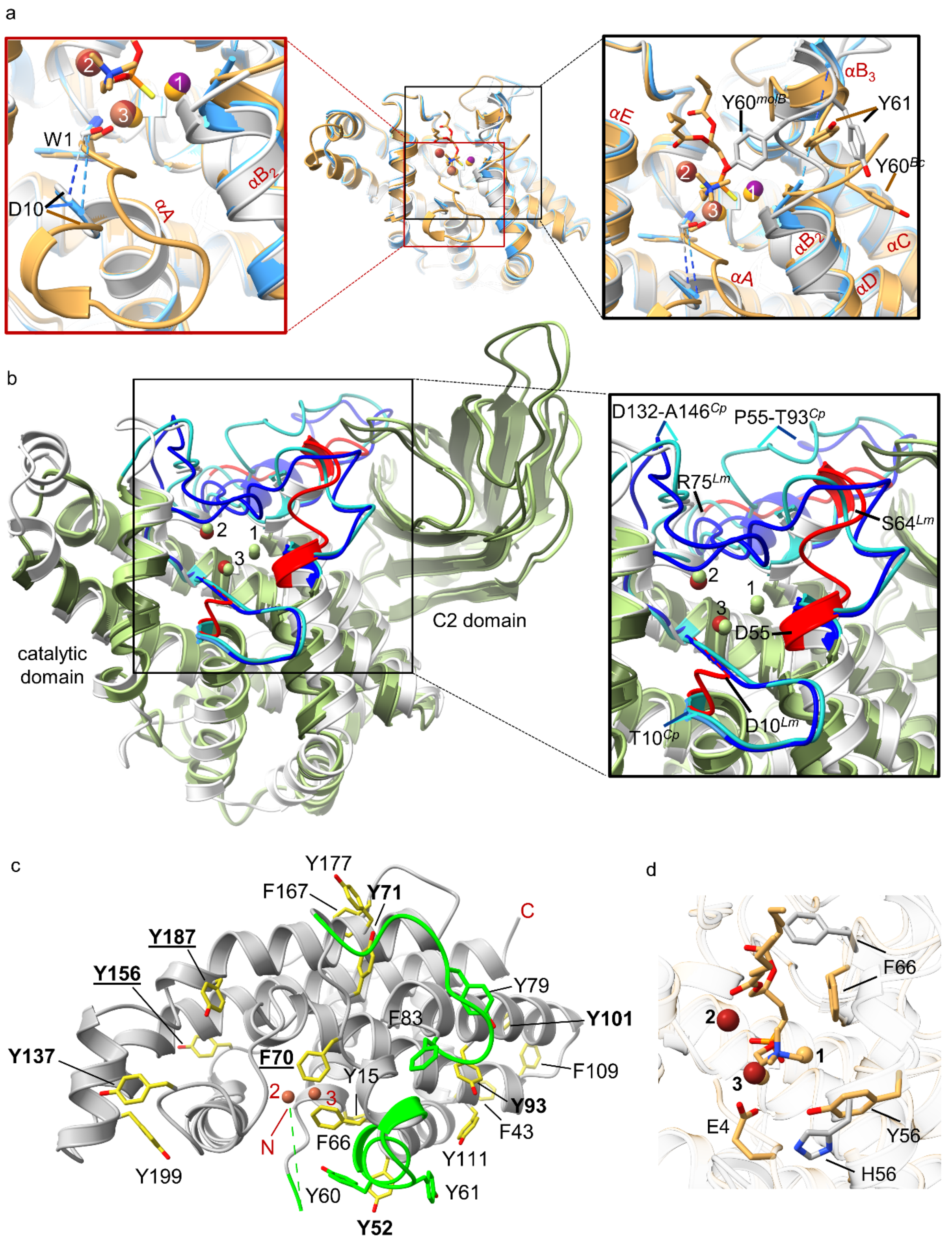

Supplementary figure 8: Structural comparison of *Lm*PC-PLC with *Bc*PC-PLC and *Cp*PC-PLC. **a** Structural alignment of *Lm*PC-PLC molA (blue), molB (grey), and *Bc*PC-PLC (orange; PDB-ID: 1P6D) with a substrate analog (orange sticks) bound to the active site of *Bc*PC-PLC. Left: Close-up of the W1 and the first loop (S2-T9) that is missing in both *Lm*PC-PLC molecules, but not in *Bc*PC-PLC. Right: A close-up of the loop D55-S64 in molB (grey) reveals occlusion in the active site by the Y60

residue. **b** Structural alignment of *Lm*PC-PLC molB (grey ribbon, violet spheres for Zn, brown spheres for Fe), open form of *Cp*PC-PLC (light green; PDB-ID: 1CA1) and closed form *Cp*PC-PLC (dark green; PDB-ID: 1GYG). Flexible loops in *Lm*PC-PLC, closed-*Cp*PC-PLC and open-*Cp*PC-PLC are shown in red, blue and cyan respectively. In the closed *Cp*PC-PLC form the loop P55-T93 interacts with membrane binding C2-domain and loop D132-A146 occludes the active site. The closed form of *Cp*PC-PLC has only two Zn^{2+} in the active site (dark green spheres), while the active one has three ions (light green spheres; two of them Cd^{2+} due to the crystallization conditions). **c** Surface exposed Tyr and Phe residues of *Lm*PC-PLC are marked green (in flexible loops) and yellow (elsewhere). Flexible loops (S2-T9, D55-S64 and R75-F83) are shown in green. Names of the structurally conserved residues between *Lm*- and *Bc*PC-PLC are shown in bold (Supplementary Fig. 1), residues conserved among all three homologues in are bold and underlined. **d** Choline binding residues E4, Y56, F66 of *Bc*PC-PLC PDB-ID: 1P6D (yellow ribbon/sticks) and *Lm*PC-PLC counterparts, molB (grey ribbon/sticks).

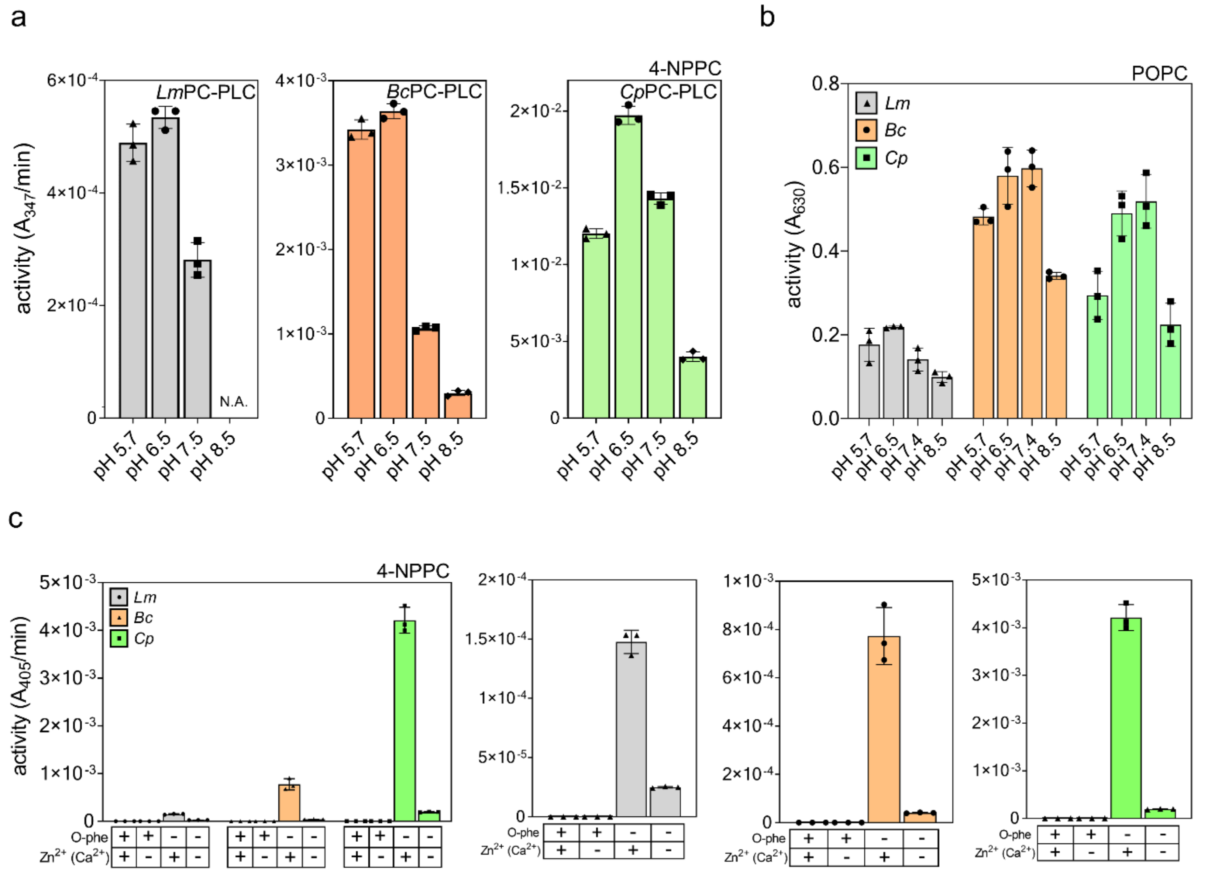

Supplementary figure 9: Enzymatic activity of *Lm*PC-PLC in comparison to *Bc*- and *Cp*-PC-PLC. **a** Activity of PC-PLC homologues (500 nM) towards 4-NPPC (1 mM) in 20 mM MES (pH 5.7, 6.5) or Tris-HCl (pH 7.4, 8.5) with 150 mM NaCl, 50 µM $ZnSO_4$, and 1 mM $CaCl_2$ (only for *Cp*). **b** Activity of PC-PLC homologues (50 nM) on 100 % POPC MLVs (4.5 mM) in 20 mM MES (pH 5.7, 6.5) or Tris-HCl pH (7.4, 8.5), 150 mM NaCl, 50 µM $ZnSO_4$, and 1 mM $CaCl_2$ (only for *Cp*). **c** Left panel: Enzymatic activity of the PC-PLC homologues (500 nM) towards 4-NPPC (1 mM) with 50 µM $ZnSO_4$ or without (control) $ZnSO_4$ and $CaCl_2$ (only for *Cp*, 1 mM) and with or without O-phe addition (1 mM). Right: the three panels are individually enlarged from the left panel to better resolve differences under various conditions used. Buffer was 20 mM MES pH 6.5, 150 mM NaCl, 0 or 50 µM $ZnSO_4$. n=3 independent experiments. Data are presented as mean values ± SEM. Source data are provided as a Source Data file.

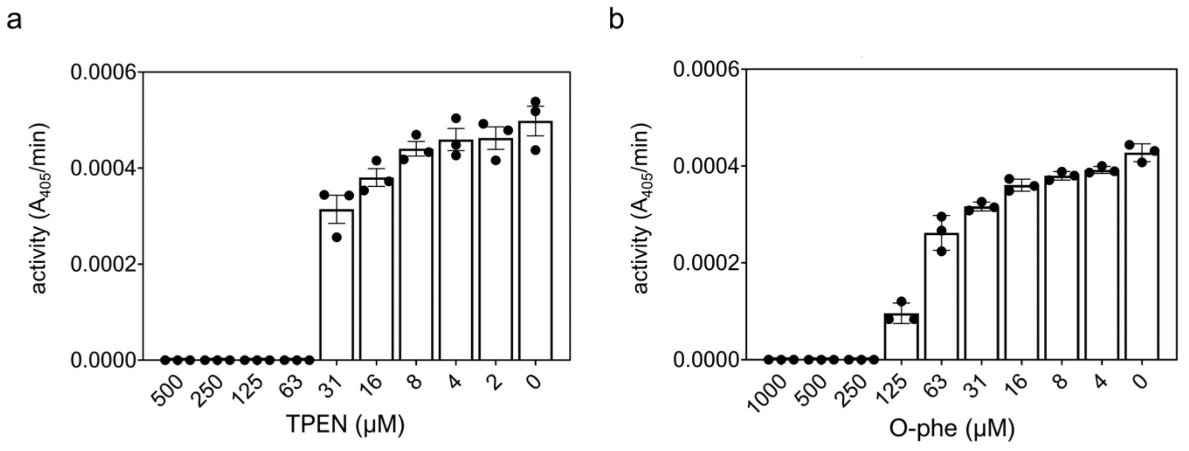

Supplementary figure 10: Comparison of inhibition of *Lm*PC-PLC by TPEN or O-phe. Activity of *Lm*PC-PLC (500 nM) towards 4-NPPC (1 mM) at different concentrations of **a** TPEN and **b** O-phe. Buffer was 20 mM MES pH 6.5, 150 mM NaCl, 50 µM $ZnSO_4$. n=3 independent experiments. Data are presented as mean values ± SEM. Source data are provided as a Source Data file.

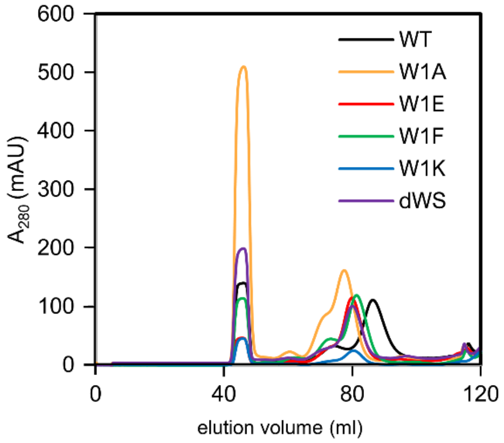

Supplementary figure 11: Gel filtration of W1 mutants of *Lm*PC-PLC. SEC analysis (Tricorn 16/600 Superdex 75 pg) of W1 mutants in comparison to the *Lm*PC-PLC WT at pH 8.5 (Tris-HCl), 500 mM NaCl.

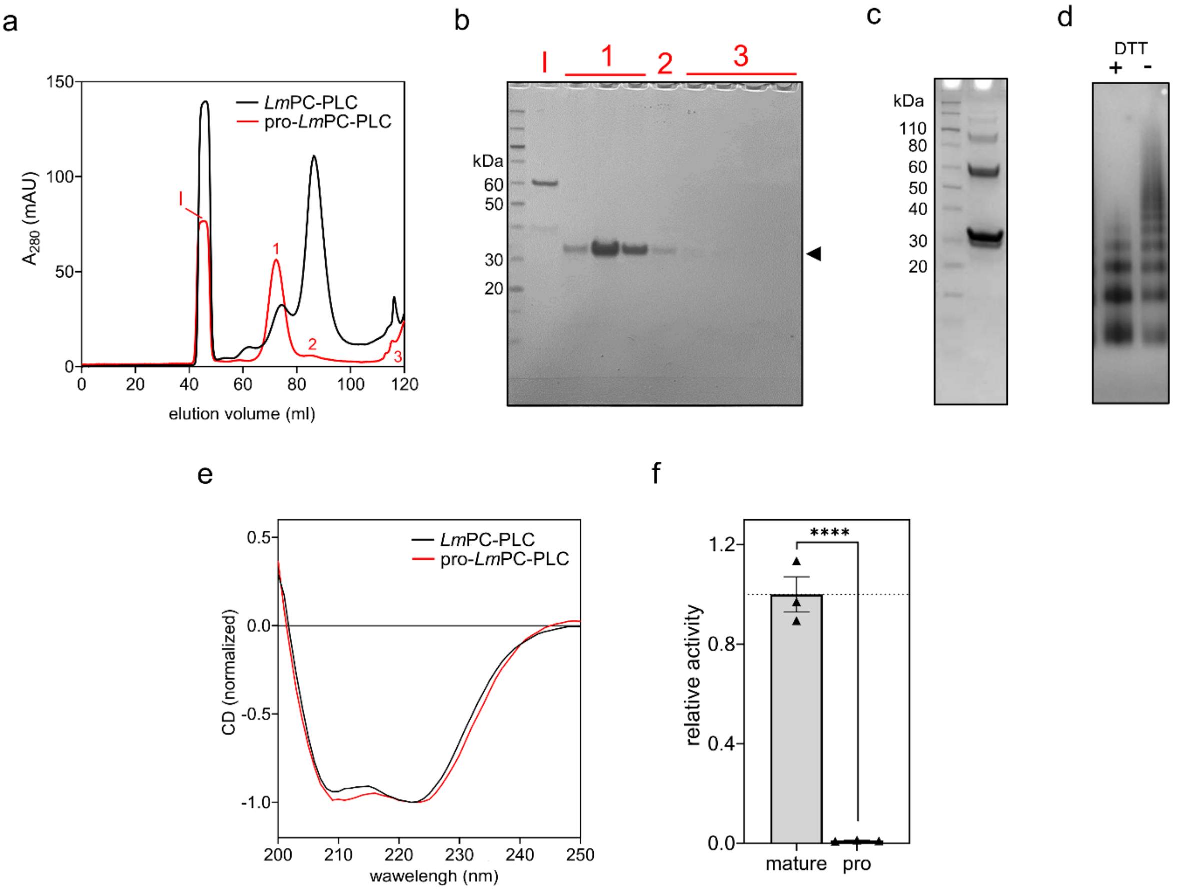

Supplementary figure 12: Characterization of pro-*Lm*PC-PLC. a SEC analysis (Tricorn 16/600 Superdex 75 pg) of pro-*Lm*PC-PLC in comparison to the mature *Lm*PC-PLC at pH 8.5 (20 mM Tris-HCl), 500 NaCl. **b** SDS-PAGE of WT pro-*Lm*PC-PLC fractions after SEC analysis (bands marked with a black triangle). **c** SDS-PAGE without DTT of pro-*Lm*PC-PLC (peak 1 from the panel a) after concentrating. **d** Blue native-PAGE of the pro-*Lm*PC-PLC (concentrated peak 1, panel a), with or without DTT (20 mM). **e** CD spectra of pro-*Lm*PC-PLC (peak 1 0.2 mg/ml) and the mature WT *Lm*PC-PLC at pH 7.4. **f** Enzymatic activities of the mature *Lm*PC-PLC and pro-*Lm*PC-PLC (50 nM) towards 100 % POPC MLV (4.5 mM). Buffer used was 20 mM MES pH 6.5, 150 mM NaCl and 50 µM $ZnSO_4$. Student's t-test was performed, ns: $P > 0.05$, *: $P < 0.05$, **: $P < 0.01$, ***: $P < 0.001$, ****: $P < 0.0001$. Data are presented as mean values ± SEM. n=3 independent experiments. Source data are provided as a Source Data file.

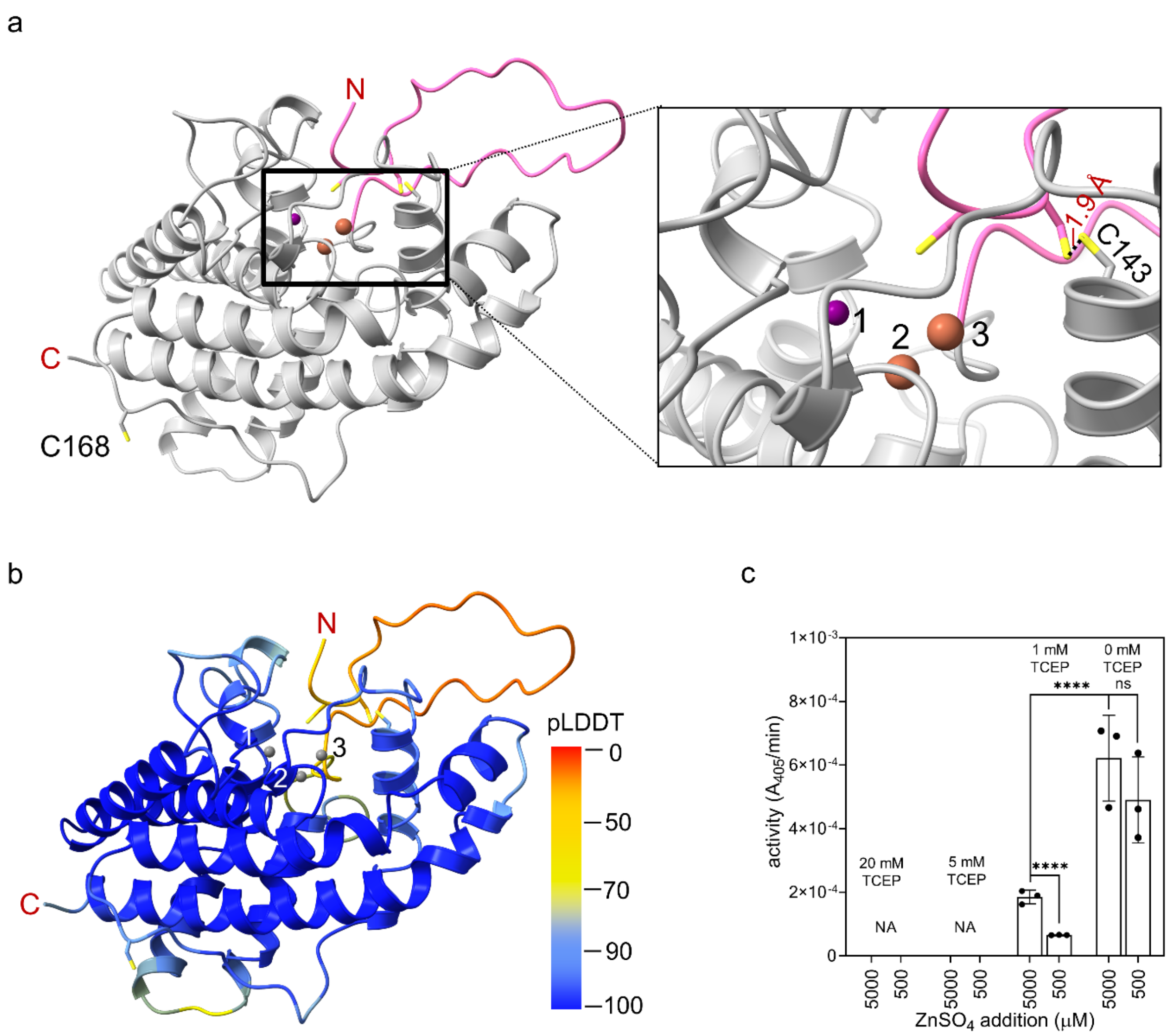

Supplementary figure 13: AF2 model of pro-*Lm*PC-PLC. a AF2[4] model of pro-*Lm*PC-PLC. Mature *Lm*PC-PLC part is in grey, propeptide is in pink ribbon. Cysteine residues are shown with sticks, and active site metal ions as spheres (Fe: brown, Zn: violet). **b** pLDDT scores for the AF2 model, active site metal ions are shown as grey spheres. **c** Effect of the reducing agent TCEP on the activity of *Lm*PC-PLC (500 nM), using 4-NPPC (1 mM) as the substrate. Concentrations of TCEP and Zn^{2+} are marked. 5 mM Zn^{2+} was used to overcome the potential Zn^{2+} binding by TCEP. Buffer used was 20 mM MES pH 6.5, 150 mM NaCl and 50 μM $ZnSO_4$. Student's t-test was performed, ns: $P > 0.05$, *: $P < 0.05$, **: $P < 0.01$, ***: $P < 0.001$, ****: $P < 0.0001$. Data are presented as mean values ± SEM. n=3 independent experiments. Source data are provided as a Source Data file.

a

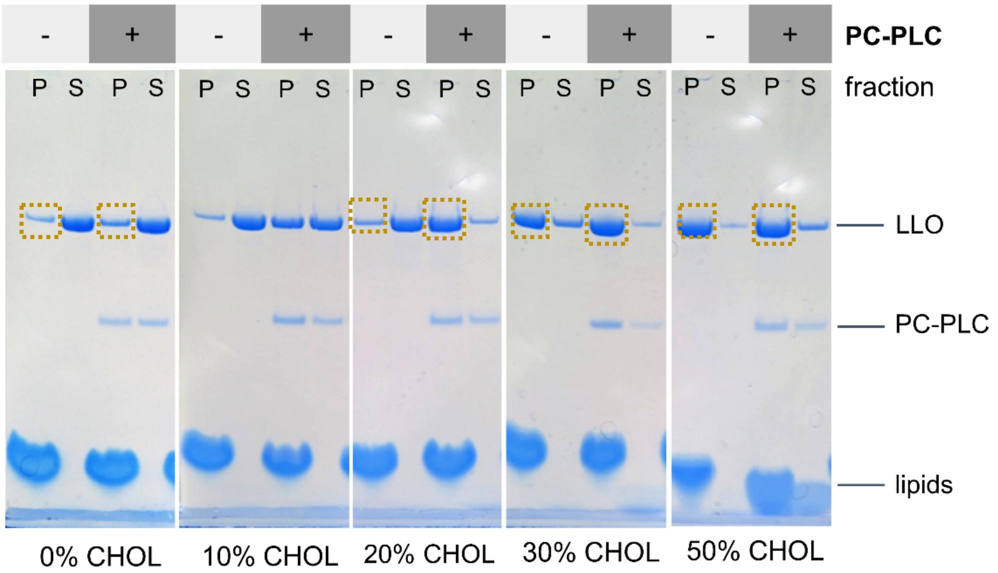

b

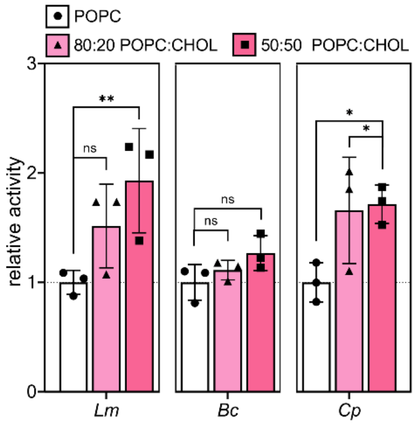

Supplementary figure 14: Effect of CHOL on LLO binding and enzymatic activity of PC-PLCs. **a** Representative SDS-PAGE of the sedimentation assay (Fig. 5a) of LLO (3 µM) to POPC:CHOL MLVs (8 mM) with various molar ratios of lipids as indicated, with or without *Lm*PC-PLC preincubation (1.4 µM). 'P' = pellet, 'S' = supernatant. Bands marked with gold brackets are the ones shown on Fig. 6. **b** Enzymatic activity of PC-PLC homologs (50 nM) on MLVs (4.5 mM) composed of different lipid mixtures of POPC and CHOL relative to the activity towards 100 % POPC. Dunnett's multiple comparisons test was performed, ns: $P > 0.05$, *: $P < 0.05$, **: $P < 0.01$, ***: $P < 0.001$, ****: $P < 0.0001$. Data are presented as mean values ± SEM. n=3 independent experiments. Buffer used (a, b) was 20 mM MES pH 6.5, 150 mM NaCl, 50 µM $ZnSO_4$, and 1 mM $CaCl_2$ (only for *Cp*) supplementation. Source data and gel replicates are provided as a Source Data file.

a

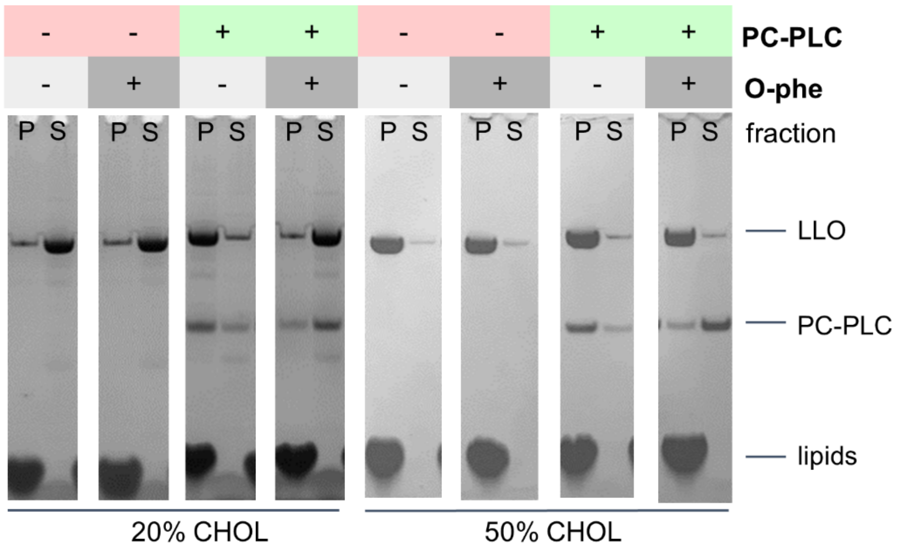

b

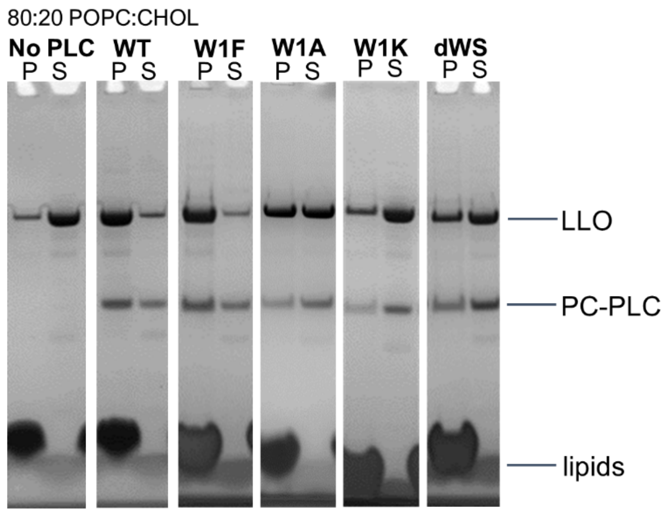

Supplementary figure 15: Full-gel displays of sedimentation assay analysis (Fig. 5c, d) of how *Lm*PC-PLC affects LLO binding to lipid membranes. a Representative SDS-PAGE of sedimentation assay of LLO (3 µM) to POPC:CHOL MLVs (8 mM) with or without *Lm*PC-PLC preincubation (1.4 µM) and with or without O-phe (1 mM). **b** Representative SDS-PAGE analysis of the sedimentation assay of LLO (3 µM) with or without preincubation with different *Lm*PC-PLC W1 mutants (1.4 µM) to POPC:CHOL 80:20 molar ratio MLVs (8 mM). Buffer was (a, b) 20 mM MES pH 6.5, 150 mM NaCl, 50 µM $ZnSO_4$. 'P' = pellet, 'S' = supernatant. Gel replicates are provided as a Source Data file.

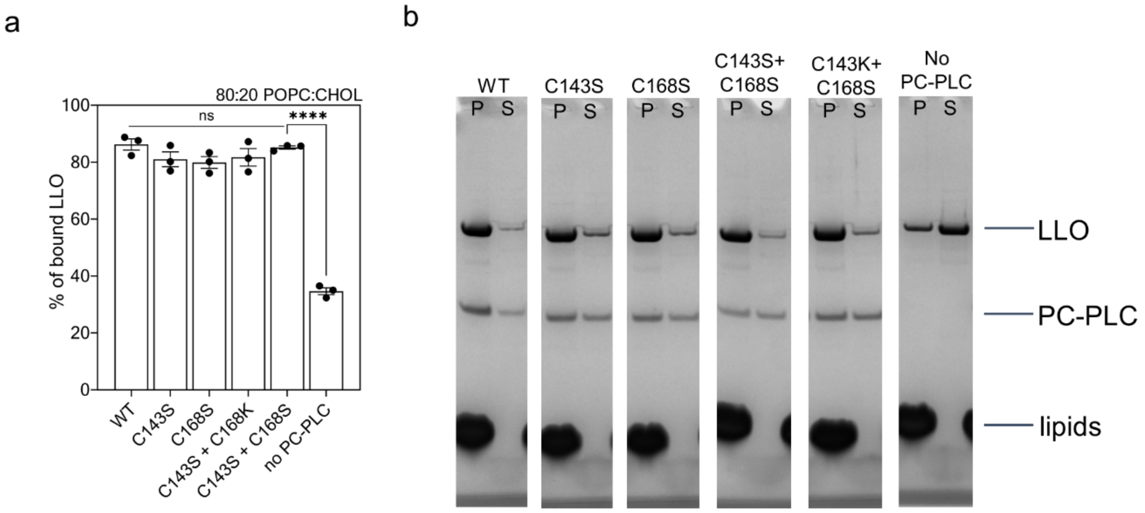

Supplementary figure 16: Effect of *Lm*PC-PLC cysteine mutants on LLO binding to membranes. **a** Sedimentation assay of LLO (3 µM) with or without preincubation of MLVs with *Lm*PC-PLC cysteine mutants (1.4 µM). MLVs (8 mM) used were composed of POPC:CHOL, molar ratio 80:20. Dunnett's multiple comparisons test was performed, ns: $P > 0.05$, *: $P < 0.05$, **: $P < 0.01$, ***: $P < 0.001$, ****: $P < 0.0001$. n=3 independent experiments. Data are presented as mean values ± SEM. **b** Representative SDS-PAGE gels of the sedimentation assay shown in the panel a. Buffer was 20 mM MES pH 6.5, 150 mM NaCl, 50 µM $ZnSO_4$. 'P' = pellet, 'S' = supernatant. Source data and gel replicates are provided as a Source Data file.

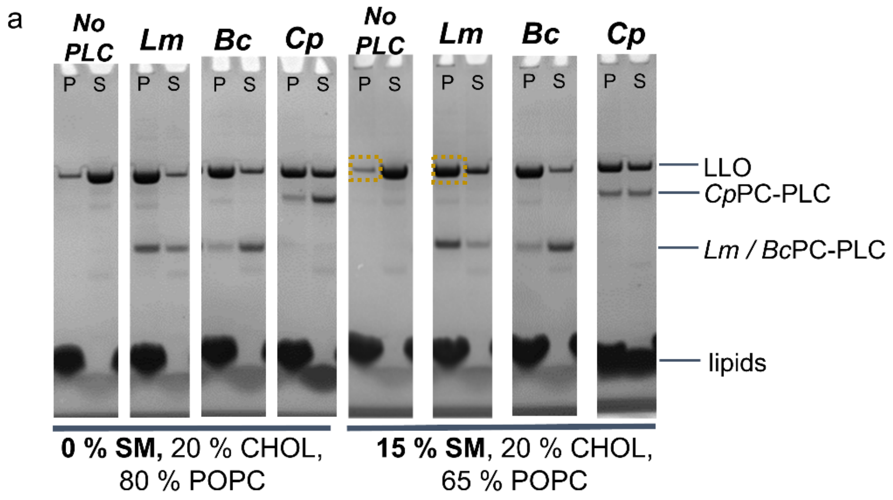

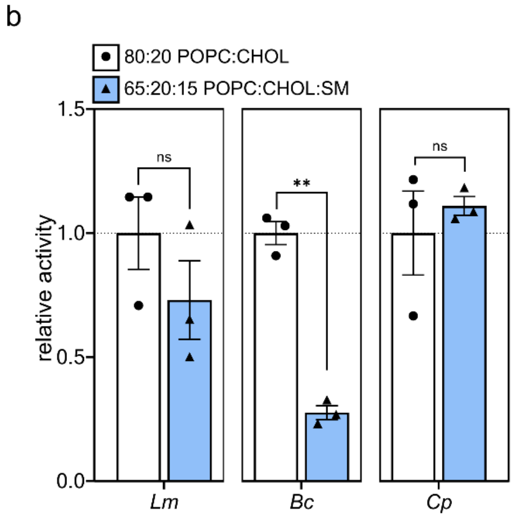

Supplementary figure 17: Full-gel displays of sedimentation assay analysis (Fig. 5e) and the effect of SM on the activity of PC-PLCs. **a** Representative SDS-PAGE analysis of the sedimentation assay of LLO (3 µM) to MLV (8 mM) containing POPC, CHOL and SM, ratios indicated on the panel, with or without the addition of different PC-PLCs (1.4 µM). Bands marked with gold brackets are the ones shown on Fig. 6. **b** Enzymatic activity of PC-PLC homologs (50 nM) towards MLVs (4.5 mM) consisting of different lipid mixtures (molar ratios) as shown on the figure. Student's t-test was performed, ns: P > 0.05, *: P < 0.05, **: P < 0.01, ***: P < 0.001, ****: P < 0.0001. Data are presented as mean values ± SEM. Buffer was (a, b) 20 mM MES pH 6.5, 150 mM NaCl, 50 µM $ZnSO_4$, and 1 mM $CaCl_2$ (only for *Cp*, 1 mM) supplementation. n=3 independent experiments. Source data and gel replicates are provided as a Source Data file.

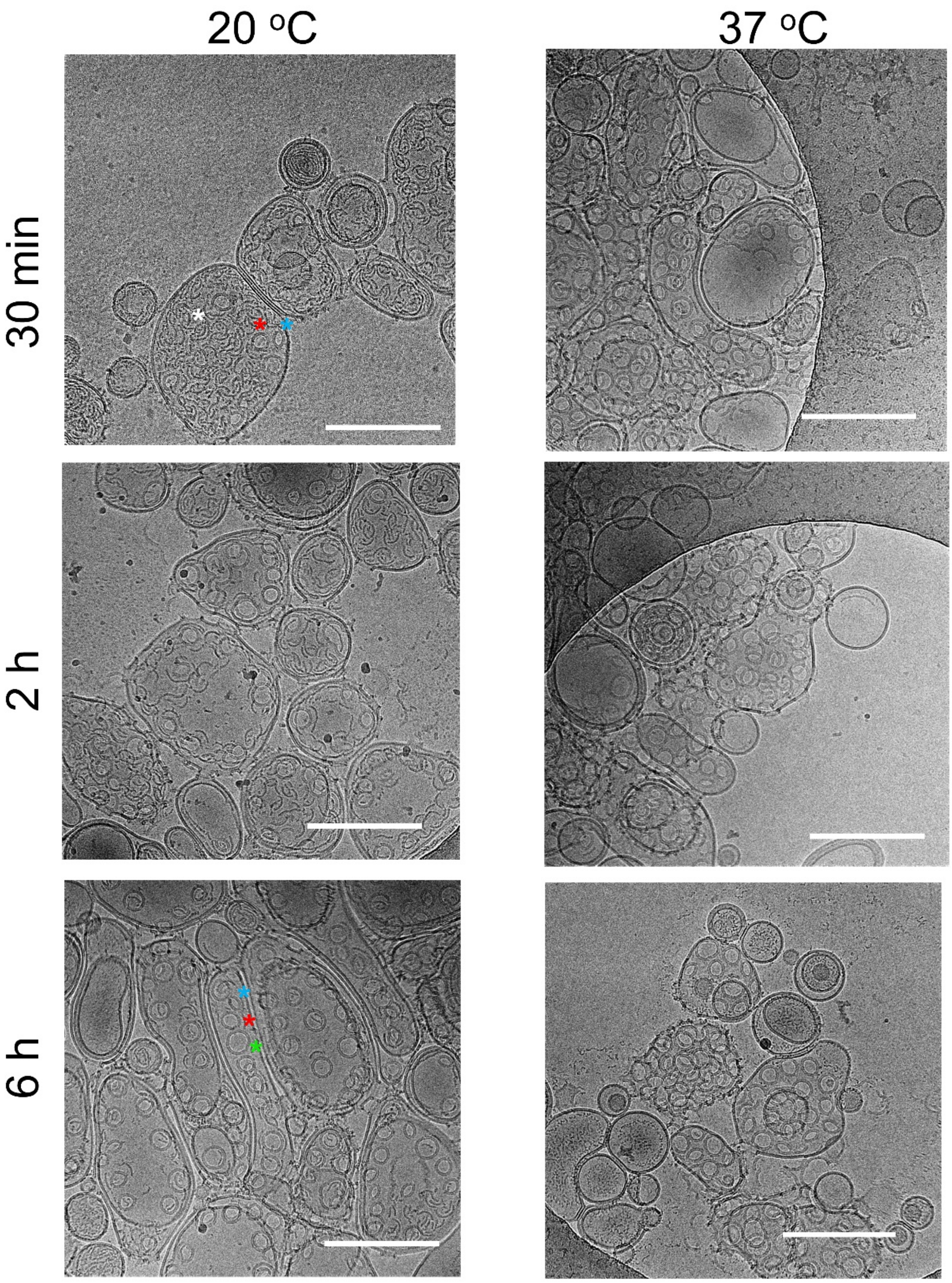

Supplementary figure 18: Cryo-EM imaging of LLO pore formation on LUVs at different times and temperatures. 73,000x magnification of 200 nm POPC:CHOL (molar ratio 1:1) LUVs (2.5 mM). Vesicles were incubated with LLO (5 µM) at different times (30 min, 2 h, 6 h) and temperatures (20 or 37 ºC) in 20 mM MES pH 6.5, 150 mM NaCl, 500 µM $ZnSO_4$. The white asterisk marks oligomeric arc formed by LLO on the membrane surface, the blue asterisk marks the LLO slit formed by two arcs inserted into the membrane, the red asterisk marks the ringed shaped LLO pore, and the green asterisk marks an expanded LLO pore. The white scale bar represents 250 nm. Two individual Cryo-EM experiments were performed with similar results.

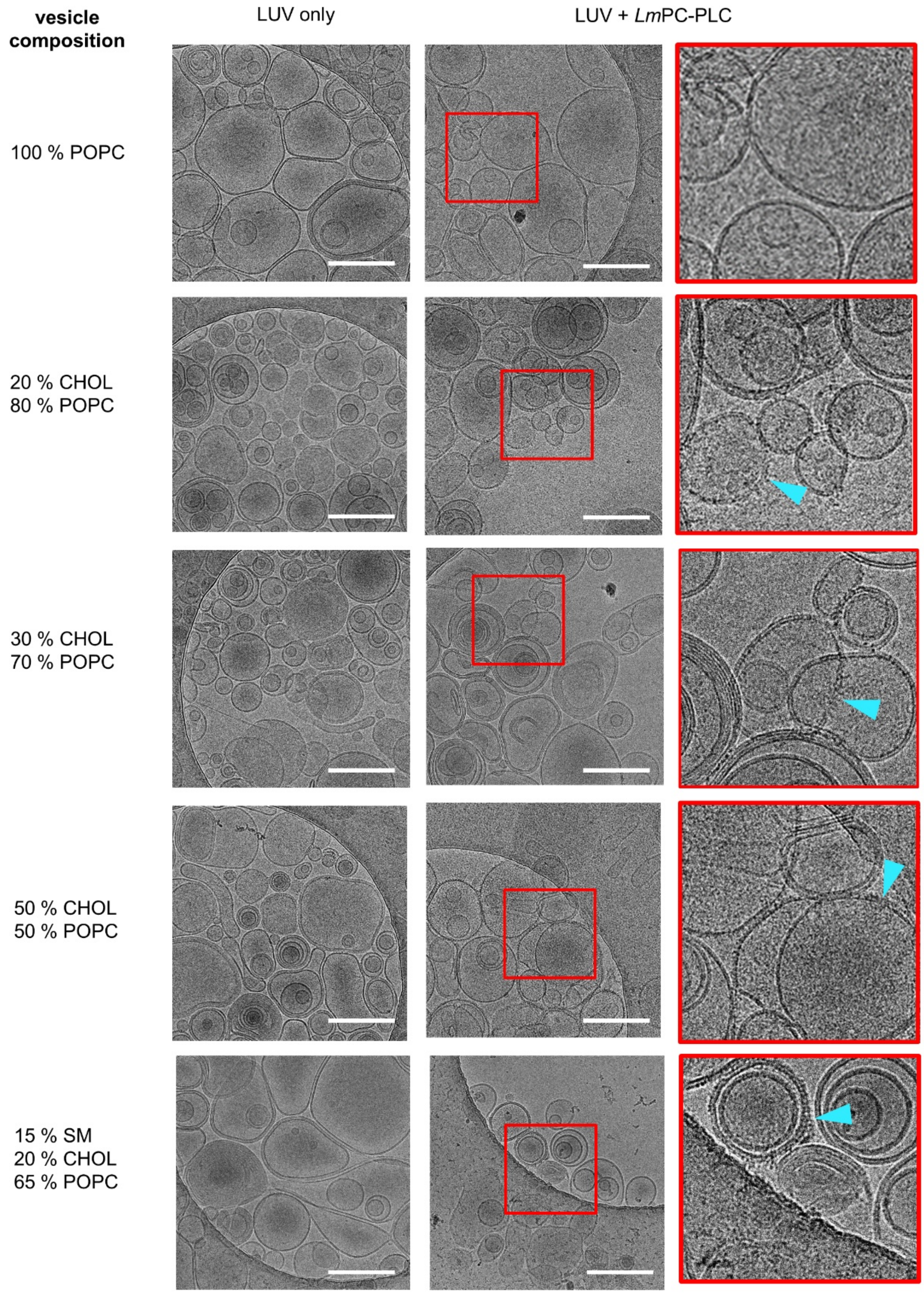

Supplementary figure 19: Cryo-EM imaging of LUVs of different lipid compositions exposed to *Lm*PC-PLC. Left: 73,000x magnification of 200 nm LUV (2.5 mM) consisting of different ratios of POPC:CHOL:SM without any protein added. Middle: LUVs (2.5 mM) incubated with *Lm*PC-PLC (5 µM) at 37 °C. Right: Close up of the area within red squares (250 nm x 250 nm). Blue arrows indicate changes of the membrane morphology. Buffer was 20 mM MES pH 6.5, 150 mM NaCl, 500 µM $ZnSO_4$. White scale bar represents 250 nm. Three individual Cryo-EM experiments were performed, except for POPC:CHOL:SM membrane, where done twice, in all cases with similar results.

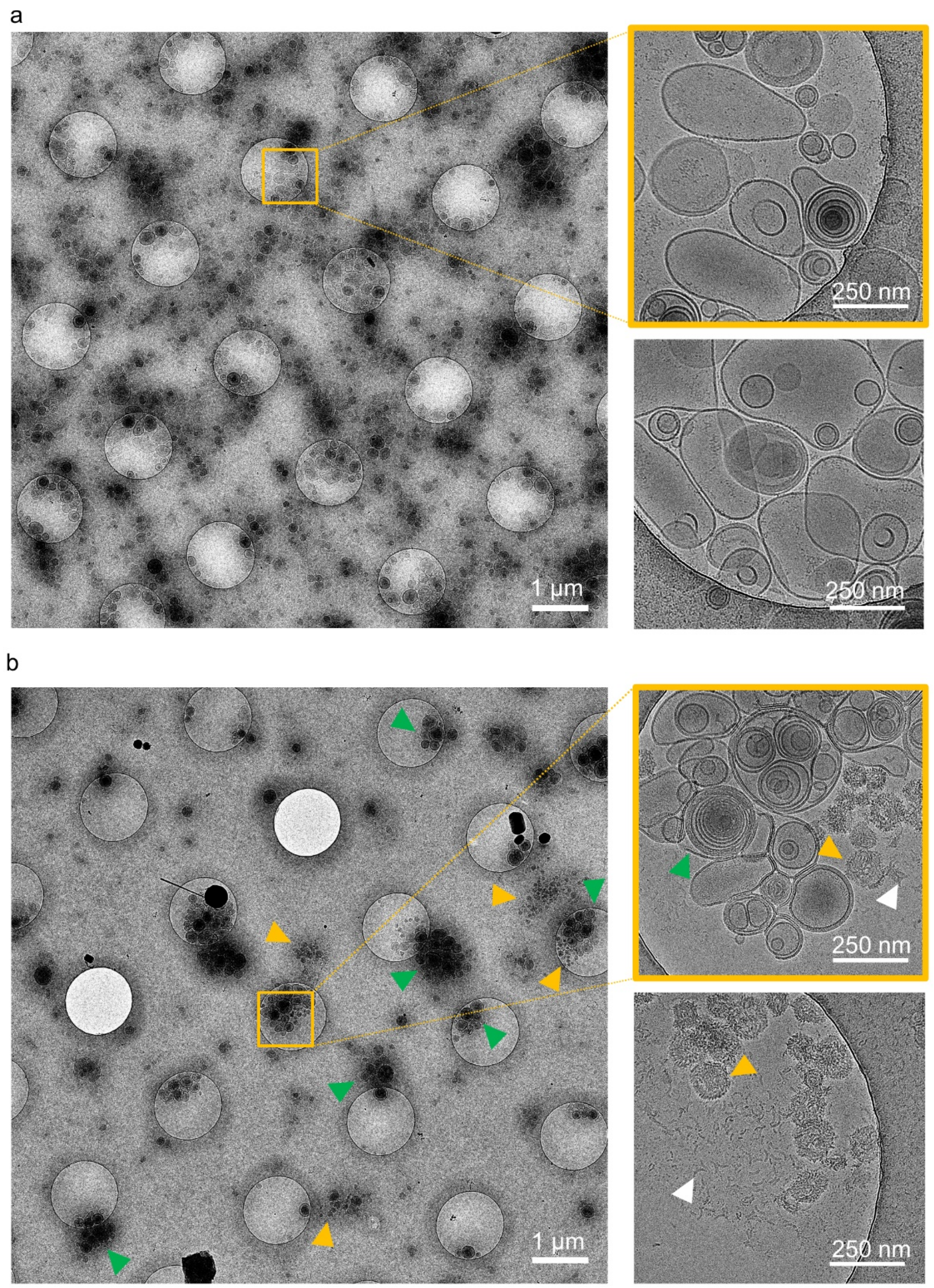

Supplementary figure 20: Cryo-EM imaging of LUVs with 20 mol % of CHOL, preincubated with *Lm*PC-PLC prior to addition of LLO. a 5,300x (left) and 73,000x (right) magnification of 200 nm LUVs composed of POPC:CHOL molar ratio 80:20 (2.5 mM) exposed only to LLO (5 µM). **b** 5,300x (left) and 73,000x (right) magnification of 200 nm LUVs of POPC:CHOL 80:20 molar ratio (2.5 mM) preincubated with *Lm*PC-PLC (5 µM) before addition of LLO (5 µM). Green arrows mark intact vesicles. Orange arrows mark LLO bound on the membrane (‘sea urchin’). White arrows mark LLO arks detached from the membrane. Buffer was 20 mM MES pH 6.5, 150 mM NaCl, 500 µM $ZnSO_4$. Three individual Cryo-EM experiments were performed with similar results.

Supplementary Table 1: Data collection and refinement statistics (from Phenix)

**Data collection**	
Wavelength (Å)	1.26
Space group	$P2_12_12_1$
a, *b*, *c* (Å)	34.91 82.92 140.82
α, β, γ (°)	90.0, 90.0, 90.0
Resolution (Å)	40.85 - 2.00 (2.072 - 2.0)
R_{meas} (%)	8.7 (66.8)
R_{pim} (%)	2.3 (18.7)
Mean of $I / \sigma I$	27.14 (4.11)
$CC_{1/2}$ (%)	100.0 (93.2)
Completeness (%)	96.7 (92.2)
Wilson B-factor (Å^2)	26.88
Total reflections	769067 (72890)
No. unique reflections▲	53292 (5186)
Redundancy	14.4 (13.7)
**Refinement**	
Resolution (Å)	40.85 - 2.00 (2.072 - 2.0)
No. unique reflections	27631 (2593)
$R_{\text{work}} / R_{\text{free}}$	0.1738 (0.1840)/ 0.2087 (0.2402)
Clash score	1.92
No. of non-hydrogen atoms	3937
Protein	3727
Ligands (2 glycerol molecules)	12
Ions	5 (1 Zn, 4 Fe)
Water	193
**B-factors (Å^2)**	
Average	30.44
Protein	30.26
Ligands	32.45
Water	33.82
**RMS deviation**	
Bond lengths (Å)	0.008
Bond angles (°)	0.91
**Ramachandran plot**	
Favored (%)	97.96 %
Allowed (%)	1.59 %
Outliers (%)▪	0.45 %

Statistics for the highest-resolution shell are shown in parentheses. Structure was solved using a single crystal of *Lm*PC-PLC. ▪Outliers in the Ramachandran plot belong to residues D76 and N77, which belong to the flexible loop R75-F83 in molB of relatively poor electron density around these two residues. ▲Friedel pairs included.

Supplementary Table 2: Occupancies and B-factors of the active site metal ions in *Lm*PC-PLC crystal structure.

molA	Occupancy	B-factors (Å^2)
Zn1	0.44	39.64
Fe2	1.00	27.00
Fe3	0.88	32.33
**molB**		
Fe2	1.00	26.59
Fe3	0.96	29.68

Supplementary Table 3: List of oligonucleotides used in the article.

Oligonucleotides used in PCR amplification and cloning

	5' - 3' sequence*	RS*
Bc^{fw}	GGTGGTTGCTCTTCCAAC**TGGAGCGCGGAAGACAAGCACAAAG**	SapI
Bc^{rev}	GGTGGTCTGCAG**TCAACGATCCCC**	PstI
Cp^{fw}	GGTGGTTGCTCTTCCAAC**TGGGATGGAAAAATTGATGGAACAGG**	SapI
Cp^{rev}	GGTGGTCTGCAGTCA**TTTTATATTATAAGTTGAATTTCCTGAAATCCAC**	PstI
Lm^{fw}	GTGGTTGCTCTTCCAAC**TGGTCCGCGGATAACCCGAC**	SapI
Lm^{rev}	GGTGGTCTGCAGTCA**TTCATTTGTTTTTTTAGACCAAA**	PstI
pro*Lm*^{fw}	GGTGGTTGCTCTTC**CAACAACGCTTG**	SapI
pro*Lm*^{rev}	GGTGGTCTGCAG**TCATTCGTTTGTCTTTTTAC**	PstI

Oligonucleotides used in PCR mutagenesis

	5' - 3' sequence*
ΔWS	GTGGTTGCTCTTCCAAC**GCGGATAACCCGACAAATAC**
W1A	GTGGTTGCTCTTCCAACGCG**TCCGCGGATAACCCGAC**
W1E	GTGGTTGCTCTTCCAACGAA**TCCGCGGATAACCCGAC**
W1F	GTGGTTGCTCTTCCAACTTT**TCCGCGGATAACCCGAC**
W1K	GTGGTTGCTCTTCCAACAAA**TCCGCGGATAACCCGAC**
D55N	**CTCAAGGAATATATGATGCG**AAT**CATAAAAATCCATATTATG**
C143S	**CCTCCAGGCTACCAC**TCT**GCATATGAAAATTAC**
C168S	G**GTAGCAAAAAGATTT**AGC**TCAGATGACGTGA**
C168K	G**ACATGGTAGCAAAAAGATTT**AAA**TCAGATGACGTGAAAGAC**

* Bold sequence shows the aligning part, underlined sequence the restriction enzyme recognition site (RS), highlighted (cyan) sequence shows mutated nucleotides. fw: forward, rev: reverse

Supplementary References

1. Krissinel, E. & Henrick, K. Secondary-structure matching (SSM), a new tool for fast protein structure alignment in three dimensions. *Acta Cryst D* **60**, 2256–2268 (2004).
2. Schrödinger, LLC. The AxPyMOL Molecular Graphics Plugin for Microsoft PowerPoint, Version 1.8. (2015).
3. Robert, X. & Gouet, P. Deciphering key features in protein structures with the new ENDscript server. *Nucleic Acids Research* **42**, W320–W324 (2014).
4. Jumper, J. *et al.* Highly accurate protein structure prediction with AlphaFold. *Nature* **596**, 583–589 (2021).
