# Supplementary material for: Structural basis for the unique molecular properties of broad-range phospholipase C from Listeria monocytogenes
Source: Nat Commun. 2023 Oct 14;14:6474. doi: 10.1038/s41467-023-42134-4 (PMC10576769; doi:10.1038/s41467-023-42134-4)
Supplement: Supplementary file 3 — Reporting Summary [file 41467_2023_42134_MOESM3_ESM.pdf]

nature portfolio

Corresponding author(s): Marjetka Podobnik

Last updated by author(s): Sep 6, 2023

Reporting Summary

Nature Portfolio wishes to improve the reproducibility of the work that we publish. This form provides structure for consistency and transparency in reporting. For further information on Nature Portfolio policies, see our Editorial Policies and the Editorial Policy Checklist.

Statistics

For all statistical analyses, confirm that the following items are present in the figure legend, table legend, main text, or Methods section.

n/a	Confirmed	
☐	☒	The exact sample size (*n*) for each experimental group/condition, given as a discrete number and unit of measurement
☐	☒	A statement on whether measurements were taken from distinct samples or whether the same sample was measured repeatedly
☐	☒	The statistical test(s) used AND whether they are one- or two-sided<br>*Only common tests should be described solely by name; describe more complex techniques in the Methods section.*
☒	☐	A description of all covariates tested
☐	☒	A description of any assumptions or corrections, such as tests of normality and adjustment for multiple comparisons
☐	☒	A full description of the statistical parameters including central tendency (e.g. means) or other basic estimates (e.g. regression coefficient) AND variation (e.g. standard deviation) or associated estimates of uncertainty (e.g. confidence intervals)
☐	☒	For null hypothesis testing, the test statistic (e.g. *F*, *t*, *r*) with confidence intervals, effect sizes, degrees of freedom and *P* value noted<br>*Give P values as exact values whenever suitable.*
☒	☐	For Bayesian analysis, information on the choice of priors and Markov chain Monte Carlo settings
☒	☐	For hierarchical and complex designs, identification of the appropriate level for tests and full reporting of outcomes
☒	☐	Estimates of effect sizes (e.g. Cohen's *d*, Pearson's *r*), indicating how they were calculated

Our web collection on statistics for biologists contains articles on many of the points above.

Software and code

Policy information about availability of computer code

Data collection	no custom code was used for data collection, all other commercial and open source software is listed and referenced in the text (incl. version number).
Data analysis	no custom code was used for data analysis, all other commercial and open source software is listed and referenced in the text and also copied bellow (incl. version number):<br>Software used: PHENIX v1.20.1, phenix.refine v1.20.1, XDSAPP v2.0, AutoSol v1.19, Coot v0.98, GraphPad Prism v8.4.2, BioTek Gen5 v3.11, GelQuant NET v1.8.2, Pymol2 v2.5.2, Chimera X v1.6.1, Astra 8 software v8.0.2.5

For manuscripts utilizing custom algorithms or software that are central to the research but not yet described in published literature, software must be made available to editors and reviewers. We strongly encourage code deposition in a community repository (e.g. GitHub). See the Nature Portfolio guidelines for submitting code & software for further information.

Data

Policy information about availability of data

All manuscripts must include a data availability statement. This statement should provide the following information, where applicable:

- Accession codes, unique identifiers, or web links for publicly available datasets
- A description of any restrictions on data availability
- For clinical datasets or third party data, please ensure that the statement adheres to our policy

The crystal structure of LmPC-PLC was deposited to wwPDB74 database under the code PDB-ID 8CQM (https://doi.org/10.2210/pdb8CQM/pdb). Raw data crystallographic data is linked to the PDB entry and is stored on XRDa database (https://doi.org/10.51093/xrd-00127). These links will be released upon publication. Source data are provided with this paper. Additional data is provided in the Supplementary information file.
Cropped gels on fig. 6 are shown in full display in the Supplementary information file (14 and 17).

Human research participants

Policy information about studies involving human research participants and Sex and Gender in Research.

Reporting on sex and gender	n/a (human participants were not included in the study)
Population characteristics	n/a
Recruitment	n/a
Ethics oversight	n/a

Note that full information on the approval of the study protocol must also be provided in the manuscript.

Field-specific reporting

Please select the one below that is the best fit for your research. If you are not sure, read the appropriate sections before making your selection.

☒ Life sciences ☐ Behavioural & social sciences ☐ Ecological, evolutionary & environmental sciences

For a reference copy of the document with all sections, see nature.com/documents/nr-reporting-summary-flat.pdf

Life sciences study design

All studies must disclose on these points even when the disclosure is negative.

Sample size	Study included only in vitro studies of purified recombinant proteins expressed in bacteria. We prepared three independent experiments (in sedimentation assay, enzyme activity assays, vesicle release experiments, as well as cryo-EM imaging). No sample size calculation was performed, since there is no population that can be referenced.
Data exclusions	No data was excluded except for calculating IC50 (Fig. 4e) where 4 data points (outliers) were excluded from the calculation, as they were determined to be outliers by the curve-fitting algorithm.
Replication	Experiments were performed with different batches of isolated proteins. Their activity and folding was confirmed and compared before including them in further experiments. Number of replications is clearly stated at every experiment shown in the article. All attempts of recreation were successful.
Randomization	Not applicable, since all the experiments were performed in vitro.
Blinding	Not applicable, since the all the experiments were performed in vitro.

Reporting for specific materials, systems and methods

We require information from authors about some types of materials, experimental systems and methods used in many studies. Here, indicate whether each material, system or method listed is relevant to your study. If you are not sure if a list item applies to your research, read the appropriate section before selecting a response.

Materials & experimental systems

n/a	Involved in the study
☒	☐ Antibodies
☒	☐ Eukaryotic cell lines
☒	☐ Palaeontology and archaeology
☒	☐ Animals and other organisms
☒	☐ Clinical data
☒	☐ Dual use research of concern

Methods

n/a	Involved in the study
☒	☐ ChIP-seq
☒	☐ Flow cytometry
☒	☐ MRI-based neuroimaging
